# Dietary inflammatory pattern and risk of hip fracture in the Nurses’ Health Study

**DOI:** 10.1007/s11657-024-01385-4

**Published:** 2024-04-24

**Authors:** Jesper Dahl, Haakon E. Meyer, Fred K. Tabung, Walter C. Willett, Kristin Holvik, Teresa T. Fung

**Affiliations:** 1 Norwegian Institute of Public Health, Skøyen, PO Box 222, 0213 Oslo, Norway; 2 Department of Community Medicine and Global Health, University of Oslo, Oslo, Norway; 3 Department of Nutrition, Harvard T.H. Chan School of Public Health, Boston, MA USA; 4 Division of Medical Oncology, Department of Internal Medicine, The Ohio State University College of Medicine, Columbus, OH USA; 5 The Ohio State University Comprehensive Cancer Center - Arthur G. James Cancer Hospital and Richard J. Solove Research Institute, Columbus, OH USA; 6 Channing Division of Network Medicine, Brigham and Women’s Hospital, Boston, MA USA; 7 Department of Nutrition, Simmons University, Boston, MA USA

**Keywords:** Hip fracture, Dietary pattern, Immune system, Postmenopause

## Abstract

***Summary*:**

Our immune system activity is impacted by what we eat and can influence fracture risk under certain conditions. In this article, we show that postmenopausal women with a pro-inflammatory dietary pattern have an increased risk of hip fracture.

**Purpose:**

The immune system influences bone homeostasis and can increase the risk of fracture under certain pro-inflammatory conditions. Immune system activity is impacted by dietary patterns. Using the empirical dietary inflammatory pattern (EDIP), we investigated whether postmenopausal women with a pro-inflammatory dietary pattern had an increased risk of hip fracture.

**Methods:**

The study population consisted of postmenopausal women participating in the Nurses’ Health Study from 1980 to 2014, who reported information on lifestyle and health, including hip fractures, on biennial questionnaires, while semiquantitative food frequency questionnaires (FFQs) were completed every fourth year. Hazard ratios (HR) for hip fracture were computed using Cox proportional hazards models, adjusting for potential confounders.

**Results:**

EDIP was calculated using intake information from the FFQ for 87,955 postmenopausal participants, of whom 2348 sustained a non-traumatic hip fracture during follow-up. After adjustment for confounders, there was a 7% increase in the risk of hip fracture per 1 SD increase in EDIP (HR 1.07, 95% CI 1.02–1.12), and the uppermost quintile had a 22% greater risk compared to the lowest (HR 1.22, 95% CI 1.06–1.40). For the separate components of the EDIP, we found that higher intakes of low-energy beverages (diet sodas) were independently associated with an increased risk of hip fracture, while higher intakes of green leafy vegetables were associated with a reduced risk.

**Conclusion:**

A pro-inflammatory dietary pattern was associated with an increased risk of hip fracture among postmenopausal women.

**Supplementary Information:**

The online version contains supplementary material available at 10.1007/s11657-024-01385-4.

## Introduction

Fracture-related burden is expected to increase significantly in the coming decades as populations continue to age [1]. To alleviate this, there is a continued focus on identifying risk factors that influence the balance between bone formation and resorption, which is vital to the continued integrity of the skeletal system. One of the factors that influences this balance is the immune system, in an interplay commonly referred to as osteoimmunology [2]. A large part of this interplay is due to a shared ancestry between certain bone and immune cells, resulting in several shared receptors. One of the known effects of this is that T-cell activation can both induce and inhibit osteoclastogenesis, depending on the cytokine profile of the activated T cells [3, 4]. T-cell activation, particularly Th17 activation, is known to induce bone degradation in inflammatory conditions such as rheumatoid arthritis [5], resulting in an increased fracture risk. It is not yet known if this pathway also significantly impacts bone health and fracture risk in the general population.

Different dietary patterns have repeatedly been shown to be associated with plasma levels of inflammatory biomarkers [6], with “Western-like” patterns generally showing an association with pro-inflammatory biomarkers. More recently developed dietary patterns have been defined using statistical methods to predict levels of specific biomarkers. One of these is the empirical dietary inflammatory pattern (EDIP), which was constructed to predict chronic systemic inflammation based on levels of interleukin-6 (IL-6), C-reactive protein (CRP), and tumor necrosis factor α receptor 2 (TNFαR2), using data from the Nurses’ Health Study (NHS) [7]. EDIP has since been shown to be associated with the development of multiple conditions, including rheumatoid arthritis in women [8].

Since IL-6 stimulates the differentiation of pro-inflammatory Th17 in favor of anti-inflammatory Treg [9], it plays an important regulatory role in the development of autoimmune disease. As Th17 is also osteoclastogenic, we would expect increased levels of IL-6 to be associated with an increased fracture risk, even outside of established autoimmune disease. We therefore hypothesized that more pro-inflammatory diets shown by higher EDIP scores would be associated with an increased risk of fracture. The aim of this study was to investigate the association between the EDIP and risk of future hip fracture in postmenopausal women.

## Study population

All individuals included in this study were participants in the NHS, which is an ongoing prospective cohort that began in 1976 [10], including 121,700 female registered nurses aged 30–55 years at enrollment. Participants in the NHS completed a biennial questionnaire on lifestyle and health, as well as a semiquantitative food frequency questionnaire (FFQ) every 4 years. The overall response rate in NHS is > 85% [11].

The current study includes data from 1980 to 2014. Follow-up began on the first questionnaire where the participant both reported having reached menopause, including surgical menopause, and had an available EDIP score.

Since fracture rates vary between ethnic groups and only < 3% of participants in the NHS are Asian or black, we chose to only include white women in the analysis. Women who reported a diagnosis of osteoporosis, cancer, or prior hip fracture at the start of follow-up were excluded from the analysis.

## Exposure—empirical dietary inflammatory pattern

The FFQ was validated and designed to assess total diet over the past 12 months [12] and included questions where the participants report their habitual frequency of consumption for specified serving sizes of more than 130 foods and beverages as well as dietary supplements. Daily energy and nutrient intakes were then calculated from the total diet. The FFQ was first distributed in 1980, then in 1984 and 1986, and every fourth year since. The current study includes FFQ data from every available questionnaire cycle during 1980–2012.

FFQ data was then used to calculate running cumulative averages of EDIP to estimate the dietary inflammatory potential among participants. It was previously constructed and validated in this study population by Tabung et al. to predict plasma levels of IL-6, CRP, and TNFαR2, by entering pre-defined food groups from FFQ data in the NHS into reduced rank regression models [7, 13].

Each of the food groups included in the EDIP is assigned a weighting which reflects their positive or inverse association with plasma levels of inflammatory markers. Of the food groups included in the EDIP, higher intakes of the following were associated with increased levels of inflammatory markers (pro-inflammatory) and contributed positively to the total EDIP score: processed meat, red meat, organ meat, fish (other than dark-meat fish), other vegetables (vegetables other than green leafy vegetables and dark-yellow vegetables), refined grains, high-energy beverages (cola and other carbonated beverages with sugar, fruit drinks), low-energy beverages (low-energy cola and other low-energy carbonated beverages), and tomatoes.

Intake of the following food groups was associated with lower levels of inflammatory markers (anti-inflammatory) and contributed inversely to total EDIP score: beer, wine, tea, coffee, dark-yellow vegetables (carrots, yellow squash, and sweet potatoes), green leafy vegetables, snacks, fruit juice, and pizza.

A higher EDIP score will reflect a more pro-inflammatory diet, while a lower score will reflect an anti-inflammatory diet. EDIP scores were calculated for each questionnaire cycle and adjusted for total energy intake using the residual method [14].

## Endpoint—hip fractures

Occurrence of hip fractures and dates of diagnosis were self-reported by participants in the biennial questionnaires. This also included the circumstances of the fracture, which was then used to classify the fracture as either traumatic or non-traumatic. Traumatic fractures included fractures caused by high-impact trauma such as, for example, skiing or traffic collisions. Hip fractures were also identified from death records. As all participants are health care professionals, the reporting was expected to be precise. This has been confirmed by a validation study which found that all reported fractures in a random subsample of the cohort were confirmed upon review of medical records [10]. Follow-up was censored at the first hip fracture for each individual, and only non-traumatic fractures were included as outcomes in the final analysis.

## Lifestyle characteristics

BMI for each questionnaire cycle was estimated using the self-reported height measurement at baseline and weight at that cycle. Other self-reported characteristics included smoking (never smoker, past smoker < 5 years, past 5–9 years, past 10 + years, current smoker < 15 cigarettes/day, current 15–24 cigarettes/day, current 25 + cigarettes/day), diagnosis of osteoporosis (yes/no), diagnosis of cancer (yes/no), diagnosis of diabetes (yes/no, does not differentiate between type I and type II), postmenopausal hormone use (never, past, and current), use of thiazides (yes/no), use of furosemide-like diuretics (yes/no), and use of oral anti-inflammatory steroids (yes/no).

Leisure-time physical activity was reported as hours per week for ten common activities. The number of hours per activity was then multiplied by the corresponding metabolic equivalent intensity level for the activity in question, before the total number of hours was summed to attain the number of metabolic equivalent hours per week per individual [15].

## Statistical analysis

Participants entered the analysis at the first questionnaire cycle following menopause with an available EDIP score and were followed until the date of a first non-traumatic hip fracture (event) or censored on the date of a first traumatic hip fracture, death, last questionnaire response, or June 1, 2014. Follow-up time at each 4-year cycle was excluded if participants had missing data for the most recent FFQ.

Cox proportional hazards models were used to calculate hazard ratios and 95% confidence intervals to assess the association of EDIP with hip fracture, with months since the start of the current questionnaire cycle as the timescale. All Cox models were stratified by age and questionnaire cycle to account for age and time.

The fully adjusted model additionally included the following covariates alongside EDIP: BMI (< 20, 20 to < 22, 22 to < 23, 23 to < 24, 24 to < 25, 25 to < 27, 27 to < 29, and 29 +), smoking (never smoker, past smoker < 5 years, past 5–9 years, past 10 + years, current smoker < 15 cigarettes/day, current 15–24 cigarettes/day, current 25 + cigarettes/day), metabolic equivalent hours (< 3, 3 to < 9, 9 to < 15, 15 to < 21, and 21 +), diagnosis of osteoporosis (yes/no), diagnosis of cancer (yes/no), diagnosis of diabetes (yes/no), postmenopausal hormone use (never, past, and current), use of thiazides (yes/no), use of furosemide-like diuretics (yes/no), and use of oral anti-inflammatory steroids (yes/no). Covariates were included as time-varying covariates in the adjusted models, meaning that person-time was assigned to the appropriate category for each variable at the beginning of every biennial follow-up questionnaire cycle.

Cumulative average EDIP (continuous and quintiles) were calculated at each 2-year cycle based on current EDIP and all previously available EDIP and entered as a time-varying covariate in the described Cox models [16]. Since the FFQs were only distributed every fourth year (except for 1986), the estimated EDIP from the previous 2-year cycle was used for cycles without an FFQ. Follow-up time for cycles with missing EDIP was excluded from the analysis.

In separate analyses, we examined the separate food group components of the EDIP as exposures in fully adjusted models. These analyses included models with unweighted daily servings of each food group, as well as models with weighted daily servings of each food group that used the same weights as the original version of the EDIP [7].

## Ethics

The study protocol was approved by the Institutional Review Boards of the Brigham and Women’s Hospital and the Harvard T.H. Chan School of Public Health. Completion and return of the self-administered questionnaires constituted informed consent.

## Results

Of 87,955 postmenopausal women with available EDIP data, 2348 sustained a non-traumatic hip fracture during follow-up. Baseline characteristics across quintiles of EDIP are presented in Table 1. The mean age at baseline was 55.0 years (SD 5.1 years). The median follow-up time was 22.1 years (SD 7.7 years), with a mean age at hip fracture of 75.6 years (SD 8.2 years). A higher EDIP score at baseline was associated with a lower level of physical activity, higher BMI, higher prevalence of diabetes, higher prevalence of use of thiazides, furosemide-like diuretics and oral anti-inflammatory steroids, lower prevalence of postmenopausal hormone use, and fewer events of osteoporosis during follow-up (Table 1).

**Table 1:** Characteristics at entry to follow-up

	**Quintiles of empirical dietary inflammatory pattern (EDIP) at entry to follow-up**					
	1st (*N* = 17,591)	2nd (*N* = 17,591)	3rd (*N* = 17,591)	4th (*N* = 17,591)	5th (*N* = 17,591)	*P*
Energy intake-adjusted dietary inflammatory index, mean (SD)	− 1.41 (0.67)	− 0.48 (0.15)	− 0.02 (0.12)	0.44 (0.15)	1.34 (0.60)	-
Non-traumatic hip fractures during follow-up, *n* (%)	478 (2.7)	468 (2.7)	491 (2.8)	459 (2.6)	452 (2.6)	0.720
Age, mean years (SD)	54.8 (4.8)	55.0 (5.0)	55.2 (5.2)	55.1 (5.2)	54.8 (5.3)	< 0.001
BMI, mean kg/m^2^ (SD)	24.5 (4.1)	25.0 (4.3)	25.6 (4.7)	26.2 (5.0)	27.4 (5.7)	< 0.001
Current smoker, *n* (%)	4562 (26.0)	3984 (22.6)	3560 (20.2)	3282 (18.7)	3725 (21.2)	< 0.001
Physical activity, METs (SD)	13.4 (18.4)	11.7 (16.9)	11.6 (16.7)	11.3 (16.7)	10.2 (15.9)	< 0.001
Current postmenopausal hormone use, *n* (%)	6272 (35.7)	5887 (33.5)	5993 (34.1)	6038 (34.3)	5687 (32.3)	< 0.001
Diagnosis of diabetes at baseline, *n* (%)	353 (2.0)	480 (2.7)	629 (3.6)	832 (4.7)	1526 (8.7)	< 0.001
Diagnosis of osteoporosis during follow-up, *n* (%)	7006 (39.8)	7228 (41.1)	7061 (40.1)	6952 (39.5)	6631 (37.7)	< 0.001
Diagnosis of cancer during follow-up, *n* (%)	4109 (23.4)	4033 (22.9)	3942 (22.4)	3994 (22.7)	3983 (22.6)	0.273
Use of thiazide diuretics, *n* (%)	1634 (10.0)	1761 (10.8)	2031 (12.5)	2384 (14.7)	2624 (16.3)	< 0.001
Use of furosemide-like diuretics, *n* (%)	45 (1.0)	49 (1.2)	68 (1.5)	102 (2.3)	146 (3.3)	< 0.001
Use of oral anti-inflammatory steroids, *n* (%)	58 (1.3)	58 (1.4)	69 (1.6)	79 (1.8)	96 (2.1)	0.016

In the fully adjusted model, we observed a 7% increase in the risk of hip fracture per SD increase in EDIP (HR 1.07, 95% CI 1.02–1.12, *P* = 0.009), with the uppermost quintile having a 22% increased risk of hip fracture compared to the lowest quintile (HR 1.22, 95% CI 1.06–1.40, *P* = 0.005) (Table 2). None of the other quintiles had an increased risk compared to the lowest.

**Table 2:** Risk of hip fracture by quintiles of cumulative empirical dietary inflammatory pattern (EDIP)

Cumulative EDIP	Age-adjusted HR	95% CI	Fully adjusted HR*	95% CI
First quintile	Ref		Ref	-
Second quintile	0.94	0.82–1.07	0.97	0.85–1.11
Third quintile	1.01	0.88–1.14	1.08	0.95–1.23
Fourth quintile	0.97	0.85–1.10	1.03	0.90–1.18
Fifth quintile	1.16	1.02–1.33	1.22	1.06–1.40
Continuous (per 1 SD)**	1.05	1.00–1.10	1.07	1.02–1.12

*Adjusted for BMI (< 20, 20 to < 22, 22 to < 23, 23 to < 24, 24 to < 25, 25 to < 27, 27 to < 29, and 29 +), smoking (never smoker, past smoker < 5 years, past 5–9 years, past 10 + years, current smoker < 15 cigarettes/day, current 15–24 cigarettes/day, current 25 + cigarettes/day), metabolic equivalent hours (< 3, 3 to < 9, 9 to < 15, 15 to < 21, and 21 +), diagnosis of osteoporosis (yes/no), diagnosis of cancer (yes/no), diagnosis of diabetes (yes/no), postmenopausal hormone use (never, past, and current), use of thiazides (yes/no), use of furosemide-like diuretics (yes/no), and use of oral anti-inflammatory steroids (yes/no)

**Separate model including EDIP as a continuous variable (as opposed to categorical quintiles) with one unit increase corresponding to one standard deviation increase in cumulative average EDIP

Analysis with the unweighted daily servings of each of the food groups included in the EDIP score in a separate fully adjusted model showed a 10% (HR 1.10, 95% CI 1.04–1.16) increased risk of hip fracture per increase in daily servings for low-energy beverages (diet sodas) and a 9% (HR 0.91, 95% CI 0.84–0.99) lower risk of hip fracture per daily servings increase in green leafy vegetables (Table 3). None of the other components was significantly associated with the risk of hip fracture. Similarly, analysis with the weighted food group components of the EDIP score also revealed an increased risk of hip fracture with increasing intake of low-energy beverages and a reduced risk with increasing intake of green leafy vegetables (Supplementary Table 1).

**Table 3:** Risk of hip fracture by components of the empirical dietary inflammatory pattern (daily servings*)

EDIP component	Age-adjusted HR	95% CI	Fully adjusted HR**	95% CI
Processed meat	1.02	0.84–1.24	1.02	0.84–1.23
Red meat	1.02	0.90–1.17	1.01	0.88–1.15
Organ meat	2.92	1.16–7.36	2.02	0.80–5.08
Fish^†^	0.95	0.77–1.17	1.02	0.83–1.25
Other vegetables^§^	1.00	0.92–1.09	1.04	0.96–1.14
Refined grains	1.03	0.98–1.08	1.02	0.97–1.06
High-energy beverages	1.08	1.00–1.18	1.05	0.96–1.14
Low-energy beverages	1.09	1.03–1.16	1.10	1.04–1.16
Tomatoes	0.97	0.88–1.06	1.00	0.91–1.10
Beer	1.08	0.93–1.26	0.99	0.85–1.16
Wine	0.96	0.88–1.05	0.96	0.88–1.05
Tea	1.01	0.97–1.05	1.01	0.97–1.05
Coffee	1.01	0.98–1.04	0.99	0.96–1.02
Dark-yellow vegetables^¶^	0.96	0.86–1.07	0.99	0.89–1.11
Green leafy vegetables	0.89	0.81–0.97	0.91	0.84–0.99
Snacks	1.03	0.97–1.10	1.02	0.96–1.09
Fruit juice	1.02	0.96–1.09	1.02	0.95–1.08
Pizza	0.48	0.23–0.99	0.54	0.26–1.10

*HRs for 1 unit increase in daily serving

**Adjusted for BMI (< 20, 20 to < 22, 22 to < 23, 23 to < 24, 24 to < 25, 25 to < 27, 27 to < 29, and 29 +), smoking (never smoker, past smoker < 5 years, past 5–9 years, past 10 + years, current smoker < 15 cigarettes/day, current 15–24 cigarettes/day, current 25 + cigarettes/day), metabolic equivalent hours (< 3, 3 to < 9, 9 to < 15, 15 to < 21, and 21 +), diagnosis of osteoporosis (yes/no), diagnosis of cancer (yes/no), diagnosis of diabetes (yes/no), postmenopausal hormone use (never, past, and current), use of thiazides (yes/no), use of furosemide-like diuretics (yes/no), and use of oral anti-inflammatory steroids (yes/no), as well as the other EDIP components

^†^Other than dark-meat fish

^§^Vegetables other than green leafy vegetables

^¶^Carrots, yellow squash, and sweet potatoes

## Discussion

A higher EDIP score was associated with an increased risk of hip fracture among postmenopausal women. Of the individual components that constitute the EDIP, an increased intake of low-energy beverages (diet sodas) and a reduced intake of green leafy vegetables were associated with an increased risk of hip fracture.

The observed association was in accordance with the stated hypothesis, with an increased dietary inflammatory potential showing an increased risk of hip fracture, but the magnitude of the association was small. This would suggest that an impact of dietary inflammatory potential on fracture risk in postmenopausal women is present but limited. These findings are in line with previous publications on dietary inflammatory indexes (DII) and fracture risk, which have shown a modest but significant association between increasing DII and increased risk of fracture [17].

The observed (crude) incidence of hip fracture during follow-up by quintiles of EDIP scores at baseline did not suggest a clear association between the two. Of the included covariates in the final model, we found that adjustment for BMI and diabetes had a relatively large impact on the final estimates. BMI is both protective of hip fracture while also being associated with an increased inflammatory state and the development of diabetes [18, 19], which again increases the risk of hip fracture. The level of risk and underlying mechanisms varies between the different types of diabetes, with individuals with type 1 diabetes commonly suffering from bone loss, while individuals with type 2 diabetes rather have an increased bone mineral density (BMD) that nonetheless seem to be more susceptible to fractures [20]. The vast majority of individuals with diabetes in the current study will have had type 2 diabetes, which likely explains why the highest quintile of EDIP (with the highest prevalence of diabetes at baseline) had the fewest events of osteoporosis during follow-up. In the end, BMI likely acts as both a mediating and a confounding factor in the current analysis, given its impact on dietary intake, and we have therefore chosen to include it as an adjusting covariate in the statistical models.

In the current context, EDIP is likely to represent multiple immunological pathways that affect bone integrity, including the promotion of IL-6 with subsequent bone destruction. However, given the relationship between EDIP and BMI/diabetes in the current study, as well as the previously reported associations between EDIP and metabolic syndrome [21, 22], it seems probable that some of the underlying mechanisms might be similar to those observed among individuals with type 2 diabetes. These mechanisms are not fully understood, but likely involve qualitative bone defects (reduced bone strength with normal or increased BMD, for example due to altered collagen structure) rather than quantitative ones, since individuals with type 2 diabetes commonly have an increased risk of hip fracture alongside an increased BMD [20]. Insulin is generally considered an anabolic agent in bone, with the insulinopenia typically seen among individuals with type 1 diabetes resulting in restricted osteoblast activity and potentially increased osteoclast activity [23]. It should also be noted that a higher EDIP is associated with a lower calcium intake, but given the lack of association between dietary calcium intake and fracture risk in multiple studies, we do not believe this to be an important factor [24].

Since the current study only included women, we do not know whether the association between the EDIP and risk of hip fracture is also present among men. Given the impact of reproductive health on immune activity, we would expect there to be more dynamism among women during a lifespan, but some of this dynamism should be removed with follow-up starting at menopause. Still, there is a large difference in the risk of hip fracture between men and women [25], and the underlying mechanisms differ.

The observed association was also only demonstrated for hip fractures and might not be present for other types of fracture. Given the large impact of hip fractures on both a societal and individual level, it is still likely the most important fracture type to study if the aim is to reduce overall fracture-related burden.

The only individual components of EDIP that were associated with hip fracture were low-energy beverages and green leafy vegetables. Low-energy beverages include a large number of artificially sweetened beverages that are known to act pro-inflammatory through IL-6-mediated pathways [26]. An increased intake may therefore impact bone mass through increased osteoclastogenesis. Similarly, but inversely, intake of green leafy vegetables, which is an important contributor of dietary magnesium, has been shown to reduce levels of IL-6 [27]. The point estimate for organ meat indicated a potentially strong association with hip fracture, but the confidence interval was very wide due to the low total intake in the study population. This potential association could be interesting to reevaluate in a population with a higher total intake of organ meat.

The current study includes a high level of detail with regard to both exposure and outcome, combined with long follow-up times, which provides us with a unique opportunity to study time-dependent associations. There are inherent limitations. Dietary data were self-reported, which would generally lead to measurement errors and underestimation of associations. However, both the dietary data and fracture incidence have been shown to have strong validity [10, 12]. Since the study population was restricted to health professionals of European ancestry, we also cannot assume that they accurately represent the broader population, although risk factors for fracture risk have generally been similar across population groups.

A pro-inflammatory dietary pattern was associated with an increased risk of hip fracture among postmenopausal women, although the strength of the association was limited.

## Supplementary Information

Below is the link to the electronic supplementary material.

Supplementary file1 (DOCX 104 KB)
