# Supplementary material for: Dietary inflammatory pattern and risk of hip fracture in the Nurses’ Health Study
Source: Arch Osteoporos. 2024 Apr 24;19(1):33. doi: 10.1007/s11657-024-01385-4 (PMC11043100; doi:10.1007/s11657-024-01385-4)
Supplement: Supplementary file 1 — Supplementary file1 (DOCX 104 KB) [file 11657_2024_1385_MOESM1_ESM.docx]

**Dietary inflammatory pattern and risk of hip fracture in the Nurses’ Health Study**

Jesper Dahl^1^, Haakon E Meyer^1,2^, Fred K Tabung^3,4,5^, Walter C Willett^3,6^, Kristin Holvik^1^, Teresa T Fung^3,7^

1. Norwegian Institute of Public Health, Oslo, Norway
2. Department of Community Medicine and Global Health, University of Oslo, Oslo, Norway
3. Department of Nutrition, Harvard T.H. Chan School of Public Health, Boston, MA, USA
4. Division of Medical Oncology, Department of Internal Medicine, The Ohio State University College of Medicine, Columbus, Ohio, USA
5. The Ohio State University Comprehensive Cancer Center - Arthur G. James Cancer Hospital and Richard J. Solove Research Institute, Columbus, Ohio, USA
6. Channing Division of Network Medicine, Brigham and Women's Hospital, Boston, MA, USA
7. Department of Nutrition, Simmons University, Boston, MA, USA

**Corresponding author:**

Jesper Dahl

Norwegian Institute of Public Health

PO Box 222 Skøyen

0213 Oslo

Norway

jesper.dahl@fhi.no

**Summary**

Our immune system activity is impacted by what we eat and can influence fracture risk under certain conditions. In this article we show that postmenopausal women with a pro-inflammatory dietary pattern have an increased risk of hip fracture.

**Abstract**

**Purpose**

The immune system influences bone homeostasis and can increase the risk of fracture under certain pro-inflammatory conditions. Immune system activity is impacted by dietary patterns. Using the empirical dietary inflammatory pattern (EDIP), we investigated whether postmenopausal women with a pro-inflammatory dietary pattern had an increased risk of hip fracture.

**Methods**

The study population consisted of postmenopausal women participating in the Nurses’ Health Study from 1980 to 2014, who reported information on lifestyle and health, including hip fractures, on biennial questionnaires, while semiquantitative food frequency questionnaires (FFQs) were completed every fourth year. Hazard ratios (HR) for hip fracture were computed using Cox proportional hazards models, adjusting for potential confounders.

**Results**

EDIP was calculated using intake information from the FFQ for 87 955 post-menopausal participants, of whom 2 348 sustained a non-traumatic hip fracture during follow-up. After adjustment for confounders there was a 7% increase in risk of hip fracture per 1 SD increase in EDIP (HR 1.07, 95% CI 1.02 – 1.12, and the uppermost quintile had a 22% greater risk compared to the lowest (HR 1.22, 95% CI 1.06 – 1.40). For the separate components of the EDIP, we found that higher intakes of low-energy beverages (diet sodas) were independently associated with an increased risk of hip fracture, while higher intakes of green-leafy vegetables were associated with a reduced risk.

**Conclusion**

A pro-inflammatory dietary pattern was associated with an increased risk of hip fracture among postmenopausal women.

**Introduction**

Fracture-related burden is expected to increase significantly in the coming decades as populations continue to age [1]. To alleviate this there is a continued focus on identifying risk factors that influence the balance between bone formation and resorption, which is vital to the continued integrity of the skeletal system. One of the factors that influences this balance is the immune system, in an interplay commonly referred to as osteoimmunology [2]. A large part of this interplay is due to a shared ancestry between certain bone and immune cells, resulting in several shared receptors. One of the known effects of this is that T-cell activation can both induce and inhibit osteoclastogenesis, depending on the cytokine profile of the activated T-cells [3, 4]. T-cell activation, particularly Th17 activation, is known to induce bone degradation in inflammatory conditions such as rheumatoid arthritis [5], resulting in an increased fracture risk. It is not yet known if this pathway also significantly impacts bone health and fracture risk in the general population.

Different dietary patterns have repeatedly been shown to be associated with plasma levels of inflammatory biomarkers [6], with “Western-like” patterns generally showing an association with pro-inflammatory biomarkers. More recently developed dietary patterns have been defined using statistical methods to predict levels of specific biomarkers. One of these is the empirical dietary inflammatory pattern (EDIP), which was constructed to predict chronic systemic inflammation based on levels of interleukin-6 (IL-6), C-reactive protein (CRP), and tumor necrosis factor α receptor 2 (TNFαR2), using data from the Nurses' Health Study (NHS) [7]. EDIP has since been shown to be associated with the development of multiple conditions, including rheumatoid arthritis in women [8].

Since IL-6 stimulates the differentiation of pro-inflammatory Th17 in favor of anti-inflammatory Treg [9], it plays an important regulatory role in the development of autoimmune disease. As Th17 is also osteoclastogenic, we would expect increased levels of IL-6 to be associated with an increased fracture risk, even outside of established autoimmune disease. We therefore hypothesized that more pro-inflammatory diets shown by higher EDIP scores would be associated with an increased risk of fracture. The aim of this study was to investigate the association between EDIP and risk of future hip fracture in postmenopausal women.

**Study Population**

All individuals included in this study were participants in the Nurses’ Health Study (NHS), which is an ongoing prospective cohort that began in 1976 [10], including 121 700 female registered nurses aged 30-55 years at enrollment. Participants in the NHS completed a biennial questionnaire on lifestyle and health, as well as a semiquantitative food frequency questionnaire (FFQ) every four years. The overall response rate in NHS is >85% [11].

The current study includes data from 1980 to 2014. Follow-up began on the first questionnaire where the participant both reported having reached menopause, including surgical menopause, and had an available EDIP score.

Since fracture rates vary between ethnic groups and only <3% of participants in the NHS are Asian or black, we chose to only include white women in the analysis. Women who reported a diagnosis of osteoporosis, cancer, or prior hip fracture at the start of follow-up were excluded from the analysis.

**Exposure – Empirical dietary inflammatory pattern**

The FFQ were validated and designed to assess total diet over the past 12 months [12], and included questions where the participants report their habitual frequency of consumption for specified serving sizes of more than 130 foods and beverages as well as dietary supplements. Daily energy and nutrient intakes were then calculated from the total diet. The FFQ was first distributed in 1980, then in 1984 and 1986, and every fourth year since. The current study includes FFQ data from every available questionnaire cycle during 1980-2012.

FFQ data was then used to calculate running cumulative averages of EDIP to estimate the dietary inflammatory potential among participants. It was previously constructed and validated in this study population by Tabung et al. to predict plasma levels of IL-6, CRP and TNFαR2, by entering pre-defined food groups from FFQ data in the NHS into reduced rank regression models [7, 13].

Each of the food groups included in the EDIP are assigned a weighting which reflects their positive or inverse association with plasma levels of inflammatory markers. Of the food groups included in the EDIP, higher intakes of the following were associated with increased levels of inflammatory markers (proinflammatory) and contributed positively to the total EDIP score: Processed meat, red meat, organ meat, fish (other than dark-meat fish), other vegetables (vegetables other than green-leafy vegetables and dark-yellow vegetables), refined grains, high-energy beverages (cola and other carbonated beverages with sugar, fruit drinks), low-energy beverages (low-energy cola and other low-energy carbonated beverages), and tomatoes.

Intake of the following food groups were associated with lower levels of inflammatory markers (anti-inflammatory) and contributed inversely to total EDIP score: Beer, wine, tea, coffee, dark-yellow vegetables (carrots, yellow squash and sweet potatoes), green-leafy vegetables, snacks, fruit juice and pizza.

A higher EDIP score will reflect a more pro-inflammatory diet, while a lower score will reflect an anti-inflammatory diet. EDIP scores were calculated for each questionnaire cycle, and adjusted for total energy intake using the residual method [14].

**Endpoint - Hip fractures**

Occurrence of hip fractures and dates of diagnosis were self-reported by participants in the biennial questionnaires. This also included the circumstances of the fracture, which was then used to classify the fracture as either traumatic- or non-traumatic. Traumatic fractures included fractures caused by high-impact trauma such as for example skiing or traffic collisions. Hip fractures were also identified from death records. As all participants are health care professionals the reporting was expected to be precise. This has been confirmed by a validation study which found that all reported fractures in a random subsample of the cohort were confirmed upon review of medical records [10]. Follow-up was censored at the first hip fracture for each individual, and only non-traumatic fractures were included as outcomes in the final analysis.

**Life-style characteristics**

BMI for each questionnaire cycle was estimated using the self-reported height measurement at baseline and weight at that cycle. Other self-reported characteristics included smoking (never smoker, past smoker <5 years, past 5–9 years, past 10+ years, current smoker <15 cigarettes/day, current 15–24 cigarettes/day, current 25+ cigarettes/day), diagnosis of osteoporosis (yes/no), diagnosis of cancer (yes/no), diagnosis of diabetes (yes/no, does not differentiate between type I and type II), postmenopausal hormone use (never, past, and current), use of thiazides (yes/no), use of furosemide-like diuretics (yes/no), and use of oral anti-inflammatory steroids (yes/no).

Leisure-time physical activity was reported as hours per week for 10 common activities. The number of hours per activity was then multiplied by the corresponding metabolic equivalent intensity level for the activity in question, before the total number of hours were summed to attain the number of metabolic equivalent hours per week per individual [15].

**Statistical analysis**

Participants entered the analysis at the first questionnaire cycle following menopause with an available EDIP score and were followed until the date of a first non-traumatic hip fracture (event) or censored on the date of a first traumatic hip fracture, death, last questionnaire response, or June 1 2014. Follow-up time at each 4-year cycle was excluded if participants had missing data for the most recent FFQ.

Cox proportional hazards models were used to calculate hazard ratios and 95% confidence intervals to assess the association of EDIP with hip fracture, with months since the start of the current questionnaire cycle as the time-scale. All Cox models were stratified by age and questionnaire cycle to account for age and time.

The fully-adjusted model additionally included the following covariates alongside EDIP: BMI (<20, 20 to <22, 22 to <23, 23 to <24, 24 to <25, 25 to <27, 27 to <29, and 29+), smoking (never smoker, past smoker <5 years, past 5–9 years, past 10+ years, current smoker <15 cigarettes/day, current 15–24 cigarettes/day, current 25+ cigarettes/day), metabolic equivalent hours (<3, 3 to <9, 9 to <15, 15 to <21, and 21+), diagnosis of osteoporosis (yes/no), diagnosis of cancer (yes/no), diagnosis of diabetes (yes/no), postmenopausal hormone use (never, past, and current), use of thiazides (yes/no), use of furosemide-like diuretics (yes/no), and use of oral anti-inflammatory steroids (yes/no). Covariates were included as time-varying covariates in the adjusted models, meaning that person-time was assigned to the appropriate category for each variable at the beginning of every biennial follow-up questionnaire cycle.

Cumulative average EDIP (continuous and quintiles) were calculated at each 2-year cycle based on current EDIP and all previously available EDIP, and entered as a time-varying covariate in the described Cox models[16]. Since the FFQs were only distributed every fourth year (except for 1986) the estimated EDIP from the previous 2-year cycle was used for cycles without an FFQ. Follow-up time for cycles with missing EDIP were excluded from the analysis.

In separate analyses, we examined the separate food group components of the EDIP as exposures in fully adjusted models. These analyses included models with unweighted daily servings of each food group, as well as models with weighted daily servings of each food group that used the same weights as the original version of the EDIP [7].

**Ethics**

The study protocol was approved by the Institutional Review Boards of the Brigham and Women's Hospital and the Harvard T.H. Chan School of Public Health. Completion and return of the self-administered questionnaires constituted informed consent.

**Results**

Of 87 955 postmenopausal women with available EDIP data, 2 348 sustained a non-traumatic hip fracture during follow-up. Baseline characteristics across quintiles of EDIP are presented in Table 1. Mean age at baseline was 55.0 years (SD 5.1 years). The median follow-up time was 22.1 years (SD 7.7 years), with a mean age at hip fracture of 75.6 years (SD 8.2 years). A higher EDIP score at baseline was associated with a lower level of physical activity, higher BMI, higher prevalence of diabetes, higher prevalence of use of thiazides, furosemide-like diuretics and oral anti-inflammatory steroids, lower prevalence of post-menopausal hormone use, and fewer events of osteoporosis during follow-up (Table 1).

**Table 1. Characteristics at entry to follow-up**

|  | Quintiles of empirical dietary inflammatory pattern (EDIP) at entry to follow-up | | | | |  |
| --- | --- | --- | --- | --- | --- | --- |
|  | 1st (N = 17 591) | 2nd (N = 17 591) | 3rd (N = 17 591) | 4th (N = 17 591) | 5th (N = 17 591) | P |
| Energy intake-adjusted dietary inflammatory index, mean (SD) | -1.41 (0.67) | -0.48 (0.15) | -0.02 (0.12) | 0.44 (0.15) | 1.34 (0.60) | - |
| Non-traumatic hip fractures during follow-up, n (%) | 478 (2.7) | 468 (2.7) | 491 (2.8) | 459 (2.6) | 452 (2.6) | 0.720 |
| Age, mean years (SD) | 54.8 (4.8) | 55.0 (5.0) | 55.2 (5.2) | 55.1 (5.2) | 54.8 (5.3) | <0.001 |
| BMI, mean kg/m2 (SD) | 24.5 (4.1) | 25.0 (4.3) | 25.6 (4.7) | 26.2 (5.0) | 27.4 (5.7) | <0.001 |
| Current smoker, n (%) | 4 562 (26.0) | 3 984 (22.6) | 3 560 (20.2) | 3 282 (18.7) | 3 725 (21.2) | <0.001 |
| Physical activity, METs (SD) | 13.4 (18.4) | 11.7 (16.9) | 11.6 (16.7) | 11.3 (16.7) | 10.2 (15.9) | <0.001 |
| Current postmenopausal hormone use, n (%) | 6 272 (35.7) | 5 887 (33.5) | 5 993 (34.1) | 6 038 (34.3) | 5 687 (32.3) | <0.001 |
| Diagnosis of diabetes at baseline, n (%) | 353 (2.0) | 480 (2.7) | 629 (3.6) | 832 (4.7) | 1 526 (8.7) | <0.001 |
| Diagnosis of osteoporosis during follow-up, n (%) | 7 006 (39.8) | 7 228 (41.1) | 7 061 (40.1) | 6 952 (39.5) | 6 631 (37.7) | <0.001 |
| Diagnosis of cancer during follow-up, n (%) | 4 109 (23.4) | 4 033 (22.9) | 3 942 (22.4) | 3 994 (22.7) | 3 983 (22.6) | 0.273 |
| Use of thiazide diuretics, n (%) | 1 634 (10.0) | 1 761 (10.8) | 2 031 (12.5) | 2 384 (14.7) | 2 624 (16.3) | <0.001 |
| Use of furosemide-like diuretics, n (%) | 45 (1.0) | 49 (1.2) | 68 (1.5) | 102 (2.3) | 146 (3.3) | <0.001 |
| Use of oral anti-inflammatory steroids, n (%) | 58 (1.3) | 58 (1.4) | 69 (1.6) | 79 (1.8) | 96 (2.1) | 0.016 |

In the fully adjusted model, we observed a 7% increase in risk of hip fracture per SD increase in EDIP (HR 1.07, 95% CI 1.02 – 1.12, P = 0.009), with the uppermost quintile having a 22% increased risk of hip fracture compared to the lowest quintile (HR 1.22, 95% CI 1.06 – 1.40, P = 0.005) (Table 2). None of the other quintiles had an increased risk compared to the lowest.

**Table 2. Risk of hip fracture by quintiles of cumulative empirical dietary inflammatory pattern (EDIP)**

| Cumulative EDIP | Age-adjusted HR | 95% CI | Fully-adjusted HR* | 95% CI |
| --- | --- | --- | --- | --- |
| First quintile | Ref. |  | Ref. | - |
| Second quintile | 0.94 | 0.82 – 1.07 | 0.97 | 0.85 - 1.11 |
| Third quintile | 1.01 | 0.88 – 1.14 | 1.08 | 0.95 - 1.23 |
| Fourth quintile | 0.97 | 0.85 – 1.10 | 1.03 | 0.90 - 1.18 |
| Fifth quintile | 1.16 | 1.02 – 1.33 | 1.22 | 1.06 - 1.40 |
|  |  |  |  |  |
| Continous (per 1 SD)** | 1.05 | 1.00 – 1.10 | 1.07 | 1.02 - 1.12 |

*Adjusted for BMI (<20, 20 to <22, 22 to <23, 23 to <24, 24 to <25, 25 to <27, 27 to <29, and 29+), smoking (never smoker, past smoker <5 years, past 5–9 years, past 10+ years, current smoker <15 cigarettes/day, current 15–24 cigarettes/day, current 25+ cigarettes/day), metabolic equivalent hours (<3, 3 to <9, 9 to <15, 15 to <21, and 21+), diagnosis of osteoporosis (yes/no), diagnosis of cancer (yes/no), diagnosis of diabetes (yes/no), postmenopausal hormone use (never, past, and current), use of thiazides (yes/no), use of furosemide-like diuretics (yes/no), and use of oral anti-inflammatory steroids (yes/no).

**Separate model including EDIP as a continuous variable (as opposed to categorical quintiles) with one unit increase corresponding to one standard deviation increase in cumulative average EDIP

Analysis with the unweighted daily servings of each of the food groups included in the EDIP score in a separate fully-adjusted model showed a 10% (HR 1.10, 95% CI 1.04 – 1.16) increased risk of hip fracture per increase in daily servings for low-energy beverages (diet sodas), and a 9 % (HR 0.91, 95% CI 0.84 – 1.00) lower risk of hip fracture per daily servings increase in green-leafy vegetables (Table 3). None of the other components were significantly associated with risk of hip fracture. Similarly, analysis with the weighted food group components of the EDIP score also revealed an increased risk of hip fracture with increasing intake of low-energy beverages, and a reduced risk with increasing intake of green-leafy vegetables (Supplementary table 1).

**Table 3. Risk of hip fracture by components of empirical dietary inflammatory pattern (daily servings*)**

| EDIP component | Age-adjusted HR | 95% CI | Fully-adjusted HR** | 95% CI |
| --- | --- | --- | --- | --- |
| Processed meat | 1.02 | 0.84 – 1.24 | 1.02 | 0.84 – 1.23 |
| Red meat | 1.02 | 0.90 – 1.17 | 1.01 | 0.88 – 1.15 |
| Organ meat | 2.92 | 1.16 – 7.36 | 2.02 | 0.80 – 5.08 |
| Fish^†^ | 0.95 | 0.77 – 1.17 | 1.02 | 0.83 – 1.25 |
| Other vegetables^§^ | 1.00 | 0.92 – 1.09 | 1.04 | 0.96 – 1.14 |
| Refined grains | 1.03 | 0.98 – 1.08 | 1.02 | 0.97 – 1.06 |
| High-energy beverages | 1.08 | 1.00 – 1.18 | 1.05 | 0.96 – 1.14 |
| Low-energy beverages | 1.09 | 1.03 – 1.16 | 1.10 | 1.04 – 1.16 |
| Tomatoes | 0.97 | 0.88 – 1.06 | 1.00 | 0.91 – 1.10 |
| Beer | 1.08 | 0.93 – 1.26 | 0.99 | 0.85 – 1.16 |
| Wine | 0.96 | 0.88 – 1.05 | 0.96 | 0.88 – 1.05 |
| Tea | 1.01 | 0.97 – 1.05 | 1.01 | 0.97 – 1.05 |
| Coffee | 1.01 | 0.98 – 1.04 | 0.99 | 0.96 – 1.02 |
| Dark-yellow vegetables^¶^ | 0.96 | 0.86 – 1.07 | 0.99 | 0.89 – 1.11 |
| Green-leafy vegetables | 0.89 | 0.81 – 0.97 | 0.91 | 0.84 – 0.99 |
| Snacks | 1.03 | 0.97 – 1.10 | 1.02 | 0.96 – 1.09 |
| Fruit juice | 1.02 | 0.96 – 1.09 | 1.02 | 0.95 – 1.08 |
| Pizza | 0.48 | 0.23 – 0.99 | 0.54 | 0.26 – 1.10 |

*HRs for 1 unit increase in daily serving

**Adjusted for BMI (<20, 20 to <22, 22 to <23, 23 to <24, 24 to <25, 25 to <27, 27 to <29, and 29+), smoking (never smoker, past smoker <5 years, past 5–9 years, past 10+ years, current smoker <15 cigarettes/day, current 15–24 cigarettes/day, current 25+ cigarettes/day), metabolic equivalent hours (<3, 3 to <9, 9 to <15, 15 to <21, and 21+), diagnosis of osteoporosis (yes/no), diagnosis of cancer (yes/no), diagnosis of diabetes (yes/no), postmenopausal hormone use (never, past, and current), use of thiazides (yes/no), use of furosemide-like diuretics (yes/no), and use of oral anti-inflammatory steroids (yes/no), as well as the other EDIP components.

^†^other than dark-meat fish

^§^vegetables other than green-leafy vegetables

^¶^carrots, yellow squash and sweet potatoes

**Supplementary table 1. Risk of hip fracture by components of empirical dietary inflammatory pattern (weighted score*)**

| EDIP component | Age-adjusted HR | 95% CI | Fully-adjusted HR** | 95% CI |
| --- | --- | --- | --- | --- |
| Processed meat | 1.14 | 0.36 – 3.64 | 1.11 | 0.35 – 3.54 |
| Red meat | 1.18 | 0.46 – 3.01 | 1.03 | 0.40 – 2.64 |
| Organ meat | 1649.62 | 2.75 – 988846.78 | 127.17 | 0.21 – 75691.28 |
| Fish^†^ | 0.81 | 0.35 – 1.85 | 1.07 | 0.48 – 2.39 |
| Other vegetables^§^ | 1.01 | 0.53 – 1.91 | 1.38 | 0.73 – 2.58 |
| Refined grains | 1.40 | 0.79 – 2.47 | 1.21 | 0.68 – 2.13 |
| High-energy beverages | 1.67 | 0.98 – 2.84 | 1.33 | 0.78 – 2.25 |
| Low-energy beverages | 2.59 | 1.43 – 4.69 | 2.78 | 1.56 – 4.94 |
| Tomatoes | 0.82 | 0.47 – 1.43 | 1.01 | 0.58 – 1.75 |
| Beer | 1.74 | 0.57 – 5.25 | 0.96 | 0.31 – 2.93 |
| Wine | 0.87 | 0.60 – 1.24 | 0.85 | 0.59 – 1.21 |
| Tea | 1.32 | 0.52 – 3.32 | 1.31 | 0.53 – 3.29 |
| Coffee | 1.18 | 0.83 – 1.68 | 0.85 | 0.59 – 1.21 |
| Dark-yellow vegetables^¶^ | 0.78 | 0.40 – 1.53 | 0.96 | 0.50 – 1.85 |
| Green-leafy vegetables | 0.54 | 0.33 – 0.86 | 0.62 | 0.39 – 0.99 |
| Snacks | 2.01 | 0.52 – 7.78 | 1.64 | 0.42 – 6.38 |
| Fruit juice | 1.42 | 0.47 – 4.25 | 1.30 | 0.44 – 3.90 |
| Pizza | 0.53 | 0.28 – 0.99 | 0.59 | 0.32 – 1.09 |

*HRs for 1 unit increase in score (intake of food group weighted by the beta coefficient from a linear regression model predicting a dietary pattern associated with IL-6, CRP and TNFαR2, see Tabung et. al. 2016 for details).

**Adjusted for BMI (<20, 20 to <22, 22 to <23, 23 to <24, 24 to <25, 25 to <27, 27 to <29, and 29+), smoking (never smoker, past smoker <5 years, past 5–9 years, past 10+ years, current smoker <15 cigarettes/day, current 15–24 cigarettes/day, current 25+ cigarettes/day), metabolic equivalent hours (<3, 3 to <9, 9 to <15, 15 to <21, and 21+), diagnosis of osteoporosis (yes/no), diagnosis of cancer (yes/no), diagnosis of diabetes (yes/no), postmenopausal hormone use (never, past, and current), use of thiazides (yes/no), use of furosemide-like diuretics (yes/no), and use of oral anti-inflammatory steroids (yes/no), as well as the other EDIP components.

^†^other than dark-meat fish

^§^vegetables other than green-leafy vegetables

^¶^carrots, yellow squash and sweet potatoes

**Discussion**

A higher EDIP score was associated with an increased risk of hip fracture among postmenopausal women. Of the individual components that constitute the EDIP, an increased intake of low-energy beverages (diet sodas) and a reduced intake of green-leafy vegetables were associated with an increased risk of hip fracture.

The observed association was in accordance with the stated hypothesis, with an increased dietary inflammatory potential showing an increased risk of hip fracture, but the magnitude of the association was small. This would suggest that an impact of dietary inflammatory potential on fracture risk in postmenopausal women is present but limited. These findings are in line with previous publications on dietary inflammatory indexes (DII) and fracture risk, which have shown a modest but significant association between increasing DII and increased risk of fracture [17].

The observed (crude) incidence of hip fracture during follow-up by quintiles of EDIP scores at baseline did not suggest a clear association between the two. Of the included covariates in the final model, we found that adjustment for BMI and diabetes had a relatively large impact on the final estimates. BMI is both protective of hip fracture while also being associated with an increased inflammatory state and the development of diabetes [18, 19], which again increases the risk of hip fracture. The level of risk and underlying mechanisms varies between the different types of diabetes, with individuals with type 1 diabetes commonly suffering from bone loss, while individuals with type 2 diabetes rather have an increased bone mineral density (BMD) that nonetheless seem to be more susceptible to fractures [20]. The vast majority of individuals with diabetes in the current study will have had type 2 diabetes, which likely explains why the highest quintile of EDIP (with the highest prevalence of diabetes at baseline) had the fewest events of osteoporosis during follow-up. In the end, BMI likely acts as both a mediating and a confounding factor in the current analysis, given its impact on dietary intake, and we have therefore chosen to include it as an adjusting covariate in the statistical models.

In the current context, EDIP is likely to represent multiple immunological pathways that affect bone integrity, including promotion of IL-6 with subsequent bone destruction. However, given the relationship between EDIP and BMI/diabetes in the current study, as well as the previously reported associations between EDIP and metabolic syndrome [21, 22], it seems probable that some of the underlying mechanisms might be similar to those observed among individuals with type 2 diabetes. These mechanisms are not fully understood, but likely involve qualitative bone defects (reduced bone strength with normal or increased BMD, for example due to altered collagen structure) rather than quantitative ones, since individuals with type 2 diabetes commonly have an increased risk of hip fracture alongside an increased BMD [20]. Insulin is generally considered an anabolic agent in bone, with the insulinopenia typically seen among individuals with type 1 diabetes resulting in restricted osteoblast activity and potentially increased osteoclast activity [23]. It should also be noted that a higher EDIP is associated with a lower calcium intake, but given the lack of association between dietary calcium intake and fracture risk in multiple studies we do not believe this to be an important factor [24].

Since the current study only included women, we do not know whether the association between EDIP and risk of hip fracture is also present among men. Given the impact of reproductive health on immune activity we would expect there to be more dynamism among women during a lifespan, but some of this dynamism should be removed with follow-up starting at menopause. Still, there is a large difference in risk of hip fracture between men and women [25], and the underlying mechanisms differ.

The observed association was also only demonstrated for hip fractures and might not be present for other types of fracture. Given the large impact of hip fractures on both a societal and individual level, it is still likely the most important fracture type to study if the aim is to reduce overall fracture-related burden.

The only individual components of EDIP that were associated with hip fracture were low-energy beverages and green-leafy vegetables. Low-energy beverages include a large number of artificially sweetened beverages that are known to act pro-inflammatory through IL-6 mediated pathways [26]. An increased intake may therefore impact bone mass through increased osteoclastogenesis. Similarly, but inversely, intake of green-leafy vegetables, which is an important contributor of dietary magnesium, has been shown to reduce levels of IL-6 [27]. The point estimate for organ meat indicated a potentially strong association with hip fracture, but the confidence interval was very wide due to the low total intake in the study population. This potential association could be interesting to reevaluate in a population with a higher total intake of organ meat.

The current study includes a high level of detail with regards to both exposure and outcome, combined with long follow-up times, which provides us with a unique opportunity to study time-dependent associations. There are inherent limitations. Dietary data were self-reported, which would generally lead to measurement errors and under-estimation of associations. However, both the dietary data and fracture incidence have been shown to have strong validity [10, 12]. Since the study population was restricted to health professionals of European ancestry, we also cannot assume that they accurately represent the broader population, although risk factors for fracture risk have generally been similar across population groups.

A pro-inflammatory dietary pattern was associated with an increased risk of hip fracture among post-menopausal women, although the strength of the association was limited.

**Funding**

The study was supported by grants from the Research Council of Norway, Dr. Trygve Gythfeldt og frues forskningsfond, and the US National Institute of Health (grant UM1 CA186107). None of the funding sources had any influence on study design or interpretation of results.

**Conflict of interest**

Jesper Dahl, Haakon E Meyer, Fred K Tabung, Walter C Willett, Kristin Holvik and Teresa T Fung declare that they have no conflicts of interest.

**References**

1. Borgstrom F, Karlsson L, Ortsater G, et al. (2020) Fragility fractures in Europe: burden, management and opportunities. Arch Osteoporos 15:59

2. Arron JR, Choi Y (2000) Bone versus immune system. Nature 408:535-536

3. Srivastava RK, Dar HY, Mishra PK (2018) Immunoporosis: Immunology of Osteoporosis-Role of T Cells. Frontiers in Immunology 9:657

4. Takayanagi H (2009) Osteoimmunology and the effects of the immune system on bone. Nature Reviews Rheumatology 5:667-676

5. Adamopoulos IE, Bowman EP (2008) Immune regulation of bone loss by Th17 cells. Arthritis Res Ther 10:225

6. Barbaresko J, Koch M, Schulze MB, Nothlings U (2013) Dietary pattern analysis and biomarkers of low-grade inflammation: a systematic literature review. Nutr Rev 71:511-527

7. Tabung FK, Smith-Warner SA, Chavarro JE, Wu K, Fuchs CS, Hu FB, Chan AT, Willett WC, Giovannucci EL (2016) Development and Validation of an Empirical Dietary Inflammatory Index. J Nutr 146:1560-1570

8. Sparks JA, Barbhaiya M, Tedeschi SK, Leatherwood CL, Tabung FK, Speyer CB, Malspeis S, Costenbader KH, Karlson EW, Lu B (2019) Inflammatory dietary pattern and risk of developing rheumatoid arthritis in women. Clin Rheumatol 38:243-250

9. Kimura A, Kishimoto T (2010) IL-6: regulator of Treg/Th17 balance. Eur J Immunol 40:1830-1835

10. Colditz GA, Martin P, Stampfer MJ, Willett WC, Sampson L, Rosner B, Hennekens CH, Speizer FE (1986) Validation of questionnaire information on risk factors and disease outcomes in a prospective cohort study of women. Am J Epidemiol 123:894-900

11. Bao Y, Bertoia ML, Lenart EB, Stampfer MJ, Willett WC, Speizer FE, Chavarro JE (2016) Origin, Methods, and Evolution of the Three Nurses' Health Studies. Am J Public Health 106:1573-1581

12. Salvini S, Hunter DJ, Sampson L, Stampfer MJ, Colditz GA, Rosner B, Willett WC (1989) Food-based validation of a dietary questionnaire: the effects of week-to-week variation in food consumption. Int J Epidemiol 18:858-867

13. Tabung FK, Smith-Warner SA, Chavarro JE, Fung TT, Hu FB, Willett WC, Giovannucci EL (2017) An Empirical Dietary Inflammatory Pattern Score Enhances Prediction of Circulating Inflammatory Biomarkers in Adults. J Nutr 147:1567-1577

14. Willett WC, Howe GR, Kushi LH (1997) Adjustment for total energy intake in epidemiologic studies. Am J Clin Nutr 65:1220S-1228S; discussion 1229S-1231S

15. Ainsworth BE, Haskell WL, Whitt MC, et al. (2000) Compendium of physical activities: an update of activity codes and MET intensities. Med Sci Sports Exerc 32:S498-504

16. Hu FB, Stampfer MJ, Rimm E, Ascherio A, Rosner BA, Spiegelman D, Willett WC (1999) Dietary fat and coronary heart disease: a comparison of approaches for adjusting for total energy intake and modeling repeated dietary measurements. Am J Epidemiol 149:531-540

17. Fang Y, Zhu J, Fan J, Sun L, Cai S, Fan C, Zhong Y, Li Y (2021) Dietary Inflammatory Index in relation to bone mineral density, osteoporosis risk and fracture risk: a systematic review and meta-analysis. Osteoporos Int 32:633-643

18. Wilson PW, Meigs JB, Sullivan L, Fox CS, Nathan DM, D'Agostino RB, Sr. (2007) Prediction of incident diabetes mellitus in middle-aged adults: the Framingham Offspring Study. Arch Intern Med 167:1068-1074

19. Søgaard AJ, Holvik K, Omsland TK, Tell GS, Dahl C, Schei B, Meyer HE (2016) Age and Sex Differences in Body Mass Index as a Predictor of Hip Fracture: A NOREPOS Study. Am J Epidemiol 184:510-519

20. Vestergaard P (2007) Discrepancies in bone mineral density and fracture risk in patients with type 1 and type 2 diabetes--a meta-analysis. Osteoporos Int 18:427-444

21. Soltani S, Moslehi N, Hosseini-Esfahani F, Vafa M (2018) The Association Between Empirical Dietary Inflammatory Pattern and Metabolic Phenotypes in Overweight/Obese Adults. Int J Endocrinol Metab 16:e60048

22. Pang T, Alman AC, Gray HL, Basu A, Shi L, Snell-Bergeon JK (2021) Empirical dietary inflammatory pattern and metabolic syndrome: prospective association in participants with and without type 1 diabetes mellitus in the coronary artery calcification in type 1 diabetes (CACTI) study. Nutr Res 94:1-9

23. Thrailkill KM, Lumpkin CK, Jr., Bunn RC, Kemp SF, Fowlkes JL (2005) Is insulin an anabolic agent in bone? Dissecting the diabetic bone for clues. Am J Physiol Endocrinol Metab 289:E735-745

24. Bolland MJ, Leung W, Tai V, Bastin S, Gamble GD, Grey A, Reid IR (2015) Calcium intake and risk of fracture: systematic review. BMJ 351:h4580

25. Farmer ME, White LR, Brody JA, Bailey KR (1984) Race and sex differences in hip fracture incidence. Am J Public Health 74:1374-1380

26. Lin WT, Kao YH, Sothern MS, Seal DW, Lee CH, Lin HY, Chen T, Tseng TS (2020) The association between sugar-sweetened beverages intake, body mass index, and inflammation in US adults. Int J Public Health 65:45-53

27. Chacko SA, Song Y, Nathan L, Tinker L, de Boer IH, Tylavsky F, Wallace R, Liu S (2010) Relations of dietary magnesium intake to biomarkers of inflammation and endothelial dysfunction in an ethnically diverse cohort of postmenopausal women. Diabetes Care 33:304-310
